# *Photobacterium profundum* under Pressure: A MS-Based Label-Free Quantitative Proteomics Study

**DOI:** 10.1371/journal.pone.0060897

**Published:** 2013-05-31

**Authors:** Thierry Le Bihan, Joe Rayner, Marcia M. Roy, Laura Spagnolo

**Affiliations:** 1 SynthSys, The University of Edinburgh, Edinburgh, United Kingdom; 2 Institute of Structural and Molecular Biology, University of Edinburgh, Edinburgh, United Kingdom; 3 Centre for Science at Extreme Conditions, University of Edinburgh, Edinburgh, United Kingdom; 4 Wellcome Trust Centre for Cell Biology, School of Biological Sciences, University of Edinburgh, Edinburgh, United Kingdom; University of Connecticut, United States of America

## Abstract

*Photobacterium profundum* SS9 is a Gram-negative bacterium, originally collected from the Sulu Sea. Its genome consists of two chromosomes and a 80 kb plasmid. Although it can grow under a wide range of pressures, *P. profundum* grows optimally at 28 MPa and 15°C. Its ability to grow at atmospheric pressure allows for both easy genetic manipulation and culture, making it a model organism to study piezophily. Here, we report a shotgun proteomic analysis of *P. profundum* grown at atmospheric compared to high pressure using label-free quantitation and mass spectrometry analysis. We have identified differentially expressed proteins involved in high pressure adaptation, which have been previously reported using other methods. Proteins involved in key metabolic pathways were also identified as being differentially expressed. Proteins involved in the glycolysis/gluconeogenesis pathway were up-regulated at high pressure. Conversely, several proteins involved in the oxidative phosphorylation pathway were up-regulated at atmospheric pressure. Some of the proteins that were differentially identified are regulated directly in response to the physical impact of pressure. The expression of some proteins involved in nutrient transport or assimilation, are likely to be directly regulated by pressure. In a natural environment, different hydrostatic pressures represent distinct ecosystems with their own particular nutrient limitations and abundances. However, the only variable considered in this study was atmospheric pressure.

## Introduction

The deep seas comprise approximately 70% of the Earth's biosphere. However, piezophiles (i.e. organisms that thrive at high pressure) have been less studied compared to other extremophiles. This is due to the difficulty of isolating and culturing them in a high pressure environment [1]. Understanding the biochemical mechanisms governing how they have adapted to live under high pressure may yield significant biotechnological and industrial applications [2].

An increase in hydrostatic pressure induces a reduction in cell volume, which affects biological reactions and cellular processes by altering macromolecular packing and hydration [3]. Therefore, any biological reaction responsible for positive or negative changes in cell volume will be affected by pressure. This may include: protein-protein interactions, ribosome assembly, protein folding, DNA conformation and interactions as well as protein-small molecule interactions [3].

*Photobacterium profundum* SS9 is a deep sea Gram negative bacterium that was originally isolated from an amphipod homogenate collected from a depth of 2.5 km in the Sulu Sea from the Philippines [1]. *Photobacterium profundum* is in the *Photobacterium* subgroup of the family *Vibrionaceae* and is, therefore, closely related to other studied *Vibrio* species [4] such as *Vibrio cholerae* (the etiological agent of cholera) and *Vibrio vulnificus* (responsible for some types of seafood poisoning and infection through open wounds) [5]. The genome sequence for *P. profundum* SS9 has been recently published and consists of two chromosomes and an 80 kb plasmid [6]. *P. profundum* is well adapted to high pressure and grows optimally at 28 MPa and 15°C, which defines it as being both a piezophile (i.e. thrives under high pressure conditions) and as a psychrophile, (i.e. thrives under cold conditions). Interestingly, *P. profundum* SS9 can grow over a large range of pressures from atmospheric pressure (0.1 MPa) up to 90 MPa [1]. *P. profundum* SS9's ability to grow at atmospheric pressure allows for the ease of genetic manipulation, culturing and the development of genetic toolsets, which are difficult to implement with many other piezophiles. For this reason, it has been adopted by the community as a model organism to study piezophily [1], [7], [8].

Several studies on *P. profundum* have shown drastic changes in both its gene expression and cellular morphology when pressure is shifted from 0.1 MPa (atmospheric pressure) to 28 MPa [4], [6], [9]. This is yet another reason for which *P. profundum* serves as a valuable piezo-tolerant model organism.

To date, two comparative transcriptomic studies have been performed on *P. profundum* at different pressures [4], [6]. It is common practice to study global changes in an organism in response to a given perturbation by using a transcriptomic approach (i.e. quantifying mRNA expression) as an estimation of the protein expression level. Although a transcriptomic approach is an essential tool to decipher mechanisms in response to a perturbation, several studies have shown poor correlation between the level of mRNA and proteins with the exception of the few most abundant proteins [10]–[12]. This observation highlights the complex relationship between mRNA and protein levels in a cell or organism due to either the importance of protein turnover or the presence of miRNA.

Until recently, proteome-wide analysis of organisms has been a challenge due to proteins not being easily amplified (as there is currently no PCR equivalent for proteins). Additionally, proteomics provides a direct measure of the global protein expression level within cells and, therefore, suffers from a strong bias toward the detection of highly abundant proteins. Fortunately, the development of more sensitive mass spectrometers with faster acquisition rates, combined with various fractionation strategies, now allows for the detection of low abundant proteins.

While several quantitative proteomic approaches exist, each has its own inherent limitations. For example, 2DE suffers from a small dynamic range and a bias toward specific classes of proteins [10]. SILAC, although currently a gold standard in the field of quantitative proteomics, is currently still limited to well-characterised *in vivo* models and can suffer from being long and tedious to establish in new model organism. In a similar way, ^15^N metabolic labelling suffers from a similar problem, since the medium composition has to be controlled and data analysis is still challenging due to heterogeneous ^15^N incorporation [13]. *In vitro* labelling strategies such as dimethylation introduce more complexity in the LC trace (reducing the number of proteins identified), isobaric labelling strategies can be rather expensive and some issues with iTRAQ accuracy and its precision have been documented by the work of Lilley's group [14].

Label-free quantitative proteomic approaches were established several years ago in the industrial proteomic field [15]–[17] and quite recently have emerged as credible quantitative tools by several academic research groups ([18], [19], to name a few). Recently, several relatively straightforward commercial software programs have been developed (for review see [20]). There are several advantages to a label-free quantitation. For example, there is no need to grow the organism with an expensive stable isotope, the method doesn't introduce more complexity by adding a heavy and a light component to each peptide, and theoretically there is no limitation from an experimental design point of view. This has made label-free quantitation an attractive strategy, even for small academic proteomic facilities, for the quantitation of changes in the cellular proteome resulting from a given perturbation.

Here, we report the first quantitative LC-MS label-free study to investigate *P. profundum*'s response to hydrostatic pressure changes for atmospheric pressure (0.1 MPa) and high pressure (28 MPa) conditions. We have identified a number of differentially expressed proteins involved in high pressure adaptation which have been previously reported, including dnaK (PBPRA1484) and GroEL (PBPRA3387) [3], [4].

Several proteins involved in key metabolic pathways were differentially expressed; 11 proteins involved in the glycolysis/gluconeogenesis pathway were up-regulated at high pressure. Conversely, at atmospheric pressure (0.1 MPa) several proteins involved in the oxidative phosphorylation pathway were up-regulated. These observations suggest that *P. profundum* may use either fermentation or respiration metabolism depending on its environment (poor oxygen content is typical of the deep sea, while higher oxygen levels characterize the surface zone).

Finally, several of the identified proteins are regulated directly in response to the physical impact of pressure. It is plausible that proteins involved in nutrient transport or assimilation, for example, have their level of expression directly regulated by pressure. The various ocean layers, from the Epipelagic zone (0 to 200 m deep) to the Mesopelagic and Bathypelagic zone (200 to 4000 m deep) represent completely distinct ecosystems with their own particular nutrient limitations and abundances. This is not the case in our study, where the only variable considered was pressure. Therefore, we hypothesize that atmospheric pressure serves as a sensing mechanism by which *P. profundum* can detect its position (depth) in the ocean. Increased pressure induces dramatic changes in the proteomic composition of this organism. Combined, these changes may result in both increased membrane fluidity and adaptation to altered nutrient availability.

## Materials and Methods

### 1. Materials

All chemicals were purchased from Sigma-Aldrich (UK) unless otherwise stated. Acetonitrile and water for LC-MS/MS and sample preparation were HPLC quality (Fisher, UK). Formic acid was Suprapure 98–100% (Merck, Darmstadt, Germany) and trifluoroacetic acid was 99% purity sequencing grade. Trypsin (modified, sequencing grade) was purchased from Roche Diagnostics (West Sussex, UK) All HPLC-MS connector fittings were from Upchurch Scientific or Valco (Hichrom and RESTEK, UK).

### 2. *Photobacterium profundum* culture and cell lysis

All *Photobacterium profundum* SS9 culture was performed anaerobically at 17°C in marine broth (28 g/liter 2216 medium; Difco Laboratories) supplemented with 20 mM glucose and 100 mM HEPES buffer (pH 7.5). To produce stock cultures, −80°C freezer stock of *P. profundum* SS9 was inoculated into 15 ml of marine broth at 17°C in sterile plastic tubes and allowed to grow to an OD of 1.5 at 600 nm. For the cultures to be used in the comparative proteomic study, 50 mL of marine broth was inoculated with100 μl of the stock cultures. The inoculum was then aliquotted into sterile plastic Pasteur pipettes [21]–[23] containing 6 ml of culture each, excluding air to avoid uneven hydrostatic pressure distribution and to ensure anaerobic conditions. Pasteur pipettes were then sealed with a Bunsen burner and a bag sealer. For growth at 0.1 MPa, pipettes were wrapped in aluminium foil and then incubated in a temperature-controlled room at 17°C. For high pressure growth, Pasteur pipettes were incubated at 28 MPa in a water-cooled pressure vessel 0.1–40 MPa at 17°C. Sets of *P. profundum* SS9 Pasteur pipette cultures from the same batch were grown simultaneously to stationary phase under two different pressure conditions in triplicate: 1) at high pressure (28 MPa) and 2) low pressure (0.1 MPa) for 5 days. The pipette cultures were then removed from their respective conditions and the cultures were harvested by centrifugation at 800×g for 10 min. Cell pellets were then snap-frozen and stored at −80°C.

Prior to analysis, cell pellets were defrosted on ice and 200 μl of 8 M urea was added to each pellet. Cells were disrupted with 100 mg acid-washed beads (425–600um, Sigma Uk) using a TissueLyser (Qiagen, Retsch, Germany) for 3 min at 30 Hz. Insoluble debris was removed by centrifugation (20 k×g 10 min at 4°C) and total protein was assayed using a Bradford kit (Biorad, UK).

### 3. Protein digestion and clean-up

Protein sample digestion was performed as described previously [24]. Peptide extracts were cleaned using a SupelClean C18 cartridge (Sigma-Aldrich, UK) and dried under low pressure. Peptide samples were stored at −20°C.

### 4. HPLC-MS analysis

Capillary-HPLC-MS/MS analysis was performed using an on-line system consisting of a micro-pump (1200 binary HPLC system, Agilent, UK) coupled to a hybrid LTQ-Orbitrap XL instrument (Thermo-Fisher, UK). The LTQ was controlled through Xcalibur 2.0.7. Samples were reconstituted in 10 µl of loading buffer before injection (8ul), and analyzed on a 2 hour gradient for data dependent analysis in a similar way as described previously [22].

### 5. Data Analysis

MS/MS data was searched using MASCOT Versions 2.4 (Matrix Science Ltd, UK) against the *Photobacterium profundum* subset of the NCBI protein database (January 2011 for a total of 5489 sequences) using a maximum missed-cut value of 2. Variable methionine oxidation and fixed cysteine carbamidomethylation were used in all searches; precursor mass tolerance was set to 7 ppm and MS/MS tolerance to 0.4 amu. The significance threshold (p) was set below 0.05 (MudPIT scoring). A peptide Mascot score of 20 was used in the final analysis, which corresponds to a global false discovery rate of 1.4% using a decoy database search. LC-MS label-free quantitation was performed using Progenesis (Nonlinear Dynamics, UK) as described elsewhere [25]. Protein conflict (peptides shared between different proteins) was solved as followed: conflict resulting from multiple sequence assignment to the same peak; we only used the sequence having the highest score. Conflict resulting from same peptide sequences assigned to different proteins, the assignment was singly attributed to the protein that had the highest number of peptides. Regarding the label-free quantitation, the total number of Features (i.e. intensity signal at a given retention time and m/z) was reduced to MS/MS peaks with charge of 2, 3, or 4+ and we only kept the five most intense MS/MS spectra per “Feature”. The subset of multi-charged ions (2+, 3+, 4+) was extracted from each LC-MS run and the ion intensities summed for normalization. Protein quantitation was performed as follows; for a specific protein, the associated unique peptide ions were summed to generate an abundance value. The measured protein abundances were transformed using an ArcSinH function (as the method of detection can generate a significant amount of near zero measurements for which a log transform is not ideal). The within group means were calculated to determine the fold change and the transformed data was then used to calculate the p-values using one way ANOVA. ArcSinH transformation was used only for the calculation of the p-value. Differentially expressed proteins were considered meaningful under the following conditions: Only proteins detected by two or more peptides, with an absolute ratio of at least 1.5 (i.e. 1.5 fold up-regulated or 0.667 down-regulated) and p<0.05 associated with the protein change.

Different bioinformatic analyses were performed in this study. Protein subcellular localization was determined using a combination of PSORTb v.3.0.2 (http://www.psort.org/psortb/index.html) [26] and CELLO (http://cello.life.nctu.edu.tw/) [27] in order to predict subcellular localisation of the proteins in a similar manner as presented by [28]. The protein GI number was then searched using NCBI BLAST to identify protein orthologs in better-characterized species, namely *Vibrio* and *E. coli*. The Kegg database (http://www.genome.jp/kegg/) was then used to identify pathway information and pathway enrichment was performed using Kobas v2.0 (http://kobas.cbi.pku.edu.cn/home.do) [29]. Data were converted using the latest PRIDE converter available v2.4.2 [30]. Data are available on the public data repository PRIDE (http://www.ebi.ac.uk/pride/). All data are also available in Supplementary information S1 and proteins identified with a single peptide reported in the text are detailed (MS/MS spectra and assignment) in Supplementary information S2 the Table and Spectra.

## Results and Discussion

All experiments were performed in biological triplicates [21]–[23] at 2 different pressures: 28 MPa and 0.1 MPa. After the cultures had been grown under their respective pressures (28 MPa and 0.1 MPa), they were all found to have a similar O.D. at 600 nm (*ca.* 1.5), suggesting they were all at the stationary phase of cell growth [22]. A total of 966 proteins (proteins with at least one unique peptide) were identified in this study. Of these proteins, 213 were differentially expressed between 28 MPa and 0.1 MPa, having a protein intensity ratio higher than 1.5, a p-value less than 0.05 and were identified with at least 2 unique peptides. The number of proteins being significantly down-regulated (i.e. ratio 28 MPa/0.1 MPa) with a p-value less than 0.05 and identified with at least 2 unique peptides was 168 proteins. All proteins identified in this study are reported in Supplementary information S1. Approximately 18% of the proteome was identified in this study with a likely bias toward the most highly abundant proteins.

**Figure 1:**
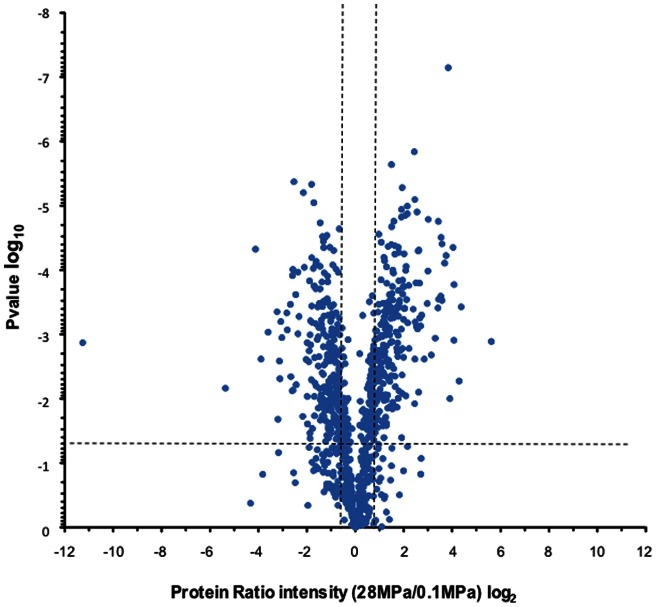
Volcano plot of the quantitative *Photobacterium profundum* proteomic data. Data statistical significance (p-value plotted in a log10 scale and calculated from a one-wayANOVA, see material and methods for details) is plotted in function of the protein ratio intensity (protein intensity at 28 MPa/protein intensity at 0.1 MPa) in a log2 scale. The horizontal dashed line shows where p-value  = 0.05 and the two vertical dashed lines separate proteins having an absolute fold-change of 1.5.

**Figure 2:**
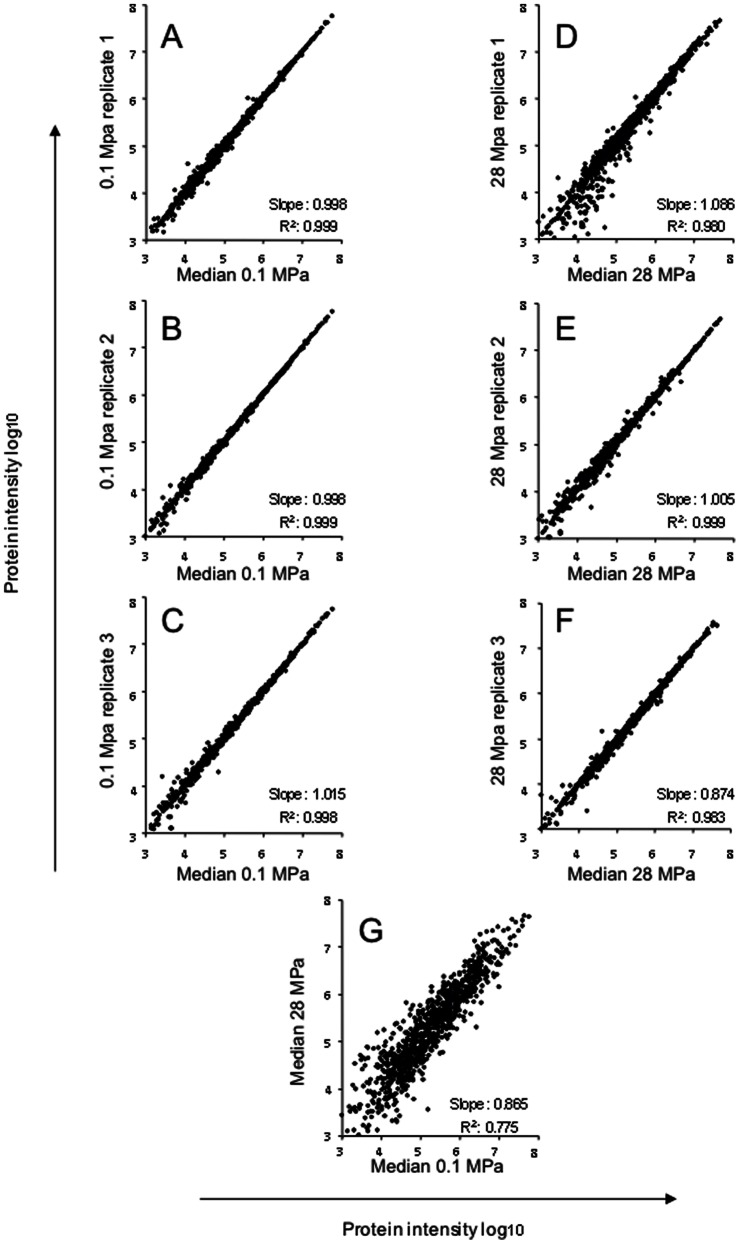
Scatter plot of the non-transformed protein intensity plotted against their median intensity group. In Fig. 2A, B, C, the biological triplicates grown at 0.1 MPa (replicates 1 to 3) is plotted in against the 0.1 MPa median intensity group. In Fig. 2D, E and F, the biological triplicates grown at 28 MPa (replicate 1 to 3) is plotted against the 28 MPa median group. In Fig. 2G, the median intensity at 28 MPa is plotted against the median intensity at 0.1 MPa. Slope and regression coefficients are also highlighted in each plot. The non- transformed protein intensity was extracted using Progenesis (see material and methods section for details).

Figure 1 illustrates a volcano plot of all p-values in function of the protein intensity ratio 28 MPa/0.1 M Pa. All values were extracted using Progenesis software. The significant number of changes detected by LC-MS are highlighted and clearly show that a global shotgun proteomic approach without intensive fractionation is sufficient to capture major changes associated with differences in growth at the 2 different pressures. In Figure 2, comparison of each individual protein's intensityis reported in function of the median intensity for each protein within a group (2A, 2B, 2C for the 0.1 MPa; 2D, 2E, 2F for the 28 MPa). Comparison of the 2 median intensity groups is shown in 2G. Normalisation was performed by Progenesis on the different LC-MS runs and shows little difference between the runs and their corresponding median, with slope varying between 0.874 to 1.086 for each sample against their respective group median.

**Figure 3:**
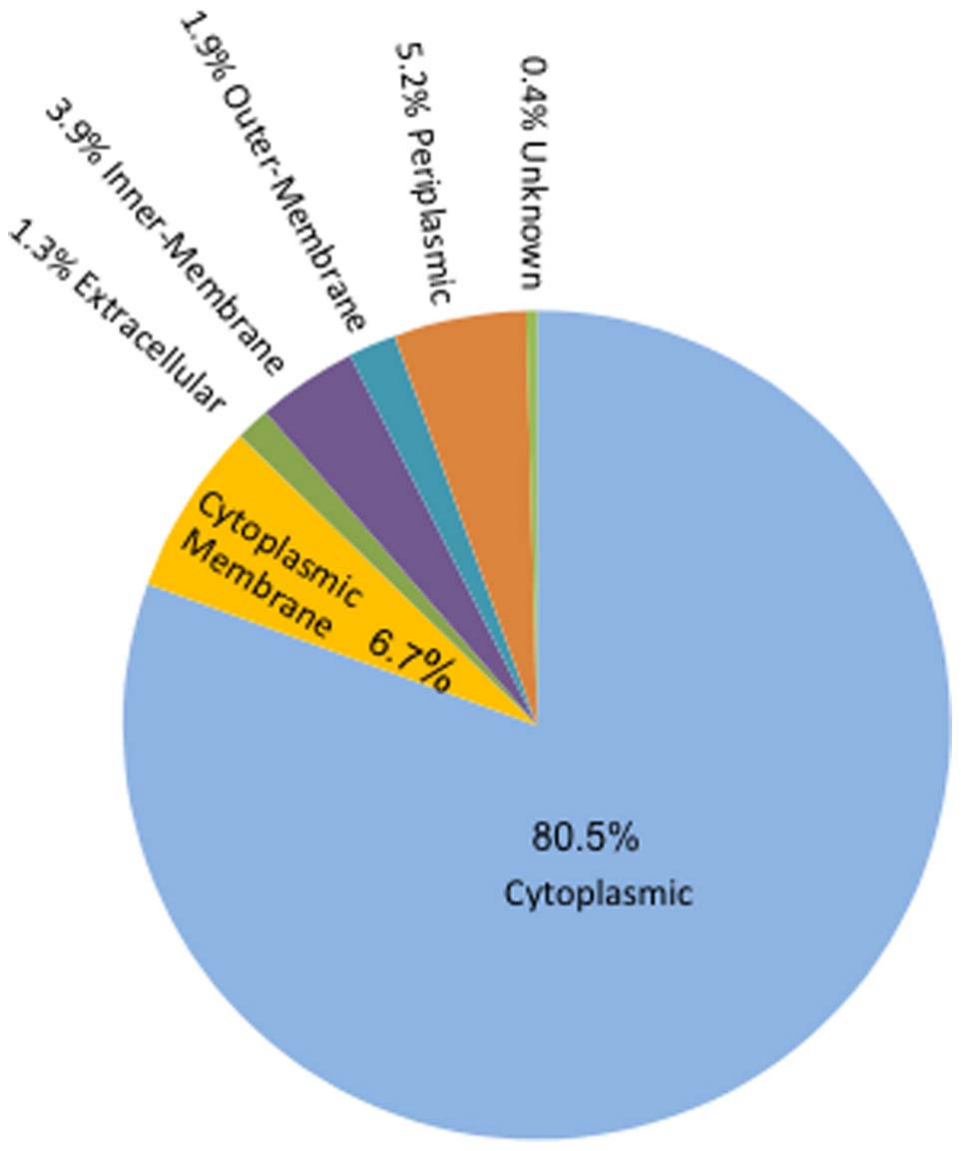
Protein localisation prediction based on PSORT (see material and method for detail). The complete set of proteins has been used for localisation distribution.

The sets of differentially expressed proteins were analyzed by pSORT and CELLO in order to establish their putative cellular localization (Figure 3). The dominant fraction of proteins identified was found to be in the cytoplasm, at 80.5% of the total of all differentially expressed proteins identified. Proteins from the cytoplasmic membrane were estimated at 6.7%% and a similar observation was made for periplasmic proteins (5.2%). Proteins having an inner-membrane localization were slightly more represented (3.9%) than the those from the outer-membrane (1.9%) and extracellular localization were estimated at 1.3% of the total proteins. A similar pattern of localization was also found for the subgroup of proteins which were reported to be significantly differentially expressed under the different pressure regimes (data not shown).

Not surprisingly, the cellular distribution reveals that the outer-membrane proteins reported to have a crucial role in pressure sensing, are poorly represented in this study. A more focused study on how *P.profundum* perceives pressure changes would benefit from a membrane enrichment strategy.

Several proteins identified in this study have been previously reported as being important for piezophilic growth (shown in Table 1). The transmembrane proteins ToxR and ToxS, for example, interact with each other and are thought to be both pressure sensing proteins as well as being involved in regulating the cellular response to pressure [31]. ToxR (PBPRA1022) was found to be down-regulated at 28 MPa, with a measured ratio of 0.25 (28 MPa/0.1 MPa) and having a p-value of 0.009, but was identified with only one unique peptide (see Supplementary information S1). ToxS (PBPRA1021P) was also one of the 966 proteins identified. However, we were unable to significantly evaluate its level of expression in relation to pressure (p-value 0.779, see Supplementary information S1). OmpL, (PBPRA0600), an outer membrane porin protein under the control of the ToxR/S complex was one of the first pressure regulated genes to be found in *Photobacterium profundum* [32]. Our data correlates well with previous studies showing that OmpL is down-regulated at 28 MPa and was identified in this study with a protein intensity ratio of 0.18 (ratio intensity 28 MPa/0.1 MPa) a p-value of 0.006 and was identified with 6 peptides).

**Table 1:** Several proteins identified in this study and been previously reported in the literature to be piezo-sensitive.

Description	Protein Id	Orthologs	Peptides used	Intensity ratio	Reference
			for quantitation^a^	28MPa/0.1MP^b^	
transcription activator ToxR	PBPRA1022	ToxR	1	0.25	Welch et al 1998
ompL_phopr porin-like protein L precursor	PBPRA0600	Ompl	6	0.18	Chi et al 1993
chaperone protein DnaJ	PBPRA0698	DnaJ	7	0.53	Campanaro et al 2005
DNA repair protein RecN	PBPRA0694	RecN	6	0.21	Vezzi et al 2005
uvrD; DNA-dependent helicase II	PBPRA3513	UvrD	8	0.38	Vezzi et al 2005
pyruvate kinase (EC:2.7.1.40); K00873	PBPRA0428		17	0.59	Vezzi et al 2005
phosphoglycerate kinase	PBPRA3131	Pgk	21	0.40	Vezzi et al 2005
glucose-6-phosphate isomerase	PBPRA3328	Pgi	20	0.62	Vezzi et al 2005

a) For a given protein, number of peptide identified in this study having a Mascot score of at least 20.

b) Protein intensity ratio at the 2 pressures in this study (28 MPa/0.1 MPa).

Other proteins with a predicted localization at the outer-membrane were also found significantly differentially regulated in function of pressure. AsmA (PBPRA1172), OmpA (PBPRB0642) as well as a lipoprotein B (PBPRA2886) were found up-regulated at 28 MPa and an outer membrane channel protein (PBPRA0450) was found down-regulated at 28 MPa. In this study, however, DnaJ (PBPRA0698) was found down-regulated at 28 MPa. Other proteins involved in piezo-sensitive mechanisms are also reported in Table 1 and compared with the literature.

Differentially expressed proteins were grouped into their respective pathways using KOBAS 2.0 (KEGG Orthology Based Annotation System). This classification was used to generate Supplementary information S3, where only those pathways showing a significant enrichment compared to *P. profundum* global genome (p-value 0.05 and less) were kept for up- and down-regulation.

Surface water and deep-sea water represent completely different physical and biochemical environments, having varying fundamental parameters. Compared to surface water, deep-sea conditions are characterized by higher pressure and the absence of light. Temperature gradients also exist, since deep-sea water is usually colder than surface water, with the exception of proximity to hot vents, where temperatures are much higher than 100°C. Other parameters that play crucial roles in biological processes include differences in oxygen, nitrate and nitrite concentrations, as well as dissolved inorganic phosphate content. Regarding oxygen levels, intermediate water (500 m–2500 m) contains less oxygen than surface and deep water. The compositional difference is partly attributed to the organic debris from the surface being decomposed while passing through this intermediate zone. Deeper sea water receives significantly less of this organic “rain” ([33] data interpreted by Copin-Montegut, 1993). Surface depletion in phosphate is attributed to a pronounced competition for scarce resources compared to the deeper zone [34]. On the other hand, the C:N ratio for particulate organic matter increases with depth and is associated with a preferential re-mineralisation of nitrogen compared to carbon during decomposition [35].

Different respiration modes driven by pressure have been previously suggested [36], [37]. In our study, putative trimethylamine-N-oxide reductase (PBPRA1468) and the anaerobic dimethyl sulfoxide reductase, subunit A (PBPRB0330) were found to be up-regulated at high pressure, which suggests a form of anaerobic respiration at 28 MPa. One consequence of trimethylamine reduction is an increase in intracellular pH. The Protein tnaA tryptophanase (PBPRA2532) (identified with 1 peptide) is also up-regulated at high pressure, which could suggest a role in counter-balancing the putative alkalinisation due to trimethylamine reduction [4]. In further regard to the up-regulation of the anaerobic respiration pathway, Periplasmic nitrate reductase (PBPRA0854) (identified with one unique peptide) and nrfA, cytochrome c552 (PBPRA1258) (identified with one unique peptide) were all found to be up-regulated under high pressure [35]. Interestingly, Cytochrome c oxidase, cbb3-type (PBPRA1834), involved in the oxidative phosphorylation pathway, was also up-regulated at high pressure. Cytochrome c oxidase cbb3-type, has a reduced proton pumping ability, but higher catalytic activity at low oxygen concentration which supports an enhanced requirement for this protein in low oxygen environments [38]. Cytochrome c oxidase bb3 type or quinol oxidase has been shown to be up-regulated under high pressure regimes [32].In contrast, a set of 6 proteins involved in the oxidative phosphorylation pathway which is typical of aerobic respiration were found up-regulated at low pressure (see Supplementary information S3 and refer to NADH dehydrogenase, PBPRA2396; cytochrome d ubiquinol oxidase subunit I (PBPRA2558), F0F1 ATP synthase subunit gamma, delta and 2 subunit alpha (PBPRA3605, PBPRA3607, PBPRA3606 and PBPRB0134, respectively). These results suggest that pressure may regulate two different modes of respiration in *Photobacterium profundum* as highlighted in the work of Kato [34].

The transport of small molecules and membrane transporters are affected by changes in hydrostatic pressure [2]. We have identified a number of significantly differentially expressed ABC transporters, which were involved in ion, sugar and amino acid transporters across the cell membrane. Specifically, we have identified different subunits of the phosphate transport ATP-binding cassette-type (ABC-type) system, such as the phosphate ABC transporter ATP-binding protein (PBPRA1391); phosphate ABC transporter, periplasmic phosphate-binding protein (PBPRA1394) as well as PhoR, phosphate regulon sensor protein (PBPRA0722) and the putative DNA-binding response regulator PhoB (PBPRA0721), which are part of a two-component system responsible for responding to phosphate limitation [39], which were also down-regulated at 28 MPa (compared to 0.1 MPa). This may both be due to changes in requirements and availability of phosphate at different sea levels and pressures, or to the transport system having evolved to function at high pressure. Phosphate transport functions less effectively at 0.1 MPa and is, therefore, required in a greater abundance by the cells. This is particularly interesting since phosphorus is a key element in marine ecosystems [40]. A similar observation has been made in this study regarding an extracellular tungstate binding protein (PBPRA1889), which was found to be up-regulated at 0.1 MPa. While we do not know the exact reason for this up-regulation, tungsten has a crucial role in the function of some oxidoreductases. Tungsten is a rare element in marine ecosystems, with the exception of hydrothermal systems [41].

We also identified a number of regulatory proteins that were significantly differentially expressed between pressure conditions and could, therefore, be new candidates for pressure-regulated gene expression. In *E. coli* the MarR (multiple antibiotic resistance regulator) family of transcriptional regulators are involved in the response to antibiotics and oxidative stresses. A MarR family regulator was also found to be present in our results showing a 7.8 fold increase from 28 MPa to 0.1 MPa (down-regulated at 28 MPa vs. 0.1 MPa) being quantified using 3 unique peptides.

A number of ribosomal proteins were differentially expressed between 28 and 0.1 MPa. Mesophilic ribosomes are one of the most pressure-sensitive structures in bacterial cells due to the particular large volume change associated with the assembly of the ribosome. An increase in pressure results in the dissociation of ribosomal subunits and the inability to form new ones [42]–[45]. A higher level of ribosomal protein subunits present at 28 MPa could allow for the existence of a constant number of assembled units independently of the pressure if the assembled structure is not favoured by high pressure. Analysis of the *P. profundum* genome identified 15 rRNAs, the largest reported for in any bacterium [6]. This, combined with the high level of variation within these rRNA operons, is thought to reflect *P. profundum* SS9's ability to rapidly respond to changes in pressure and the requirement to alter ribosomal structure in function of atmospheric pressure [46]. There were a total of 25 significantly up-regulated ribosomal proteins present in our data (see Supplementary information S3) and they represent an enrichment having a p-value of 3×10^−9^.This is one of the highest enrichment factors obtained for any group of proteins identified in this study.

Transcriptome analysis at 0.1 MPa versus 28 MPa, showed an up-regulation of DnaK, DnaJ and GroEL [6], [47]. It has been previously speculated that this could be a piezophilic response to survive shallow-water conditions when *P. profundum* is located far from the deep-sea [47]. In our study, we see that GroEL (PBPRA3387) and DnaK (PBPRA0697) are instead up-regulated at 28 MPa, while DnaJ is down-regulated. An anti-correlation between the proteomic and transcriptomic data has been previously highlighted with regards to proteins associated with the cellular stress responses in the work of Hack in 2004 [10]. This may well explain the observations made in this study. Our differing results for DnaK and DnaJ may also be due to their involvement in the very early phases of the cellular stress response. While all care was taken to harvest and freeze cells as quickly as possible, it may be that some stress response signals were activated as soon as the cell cultures were de-pressurized. Of course, this problem is intrinsic in all of the studies performed on *P. profundum* to date, and only limited to the most rapid changes in protein expression.

Specific and unique enzymes involved in the glycolysis/gluconeogenesis were identified as being differentially regulated in both of the pressure conditions being tested (a Kegg pathway diagram is presented in Supplementary information S4). Surprisingly, the enzyme involved in the phosphotransferase (PTS) system, glucose-specific IIBC component (EC:2.7.1.69) has 2 isoforms differentially expressed at each pressure(shown in yellow in Supplementary information S4). The isoform PBPRA0861 with 169aa (PTS system glucose-specific transporter subunit) is up-regulated at high pressure while the other isoform (PTS system glucose-specific transporter subunits IIBC) (PBPRA1203) with 477 aa is up-regulated at atmospheric pressure. The genes encoding both isoforms of this enzyme (EC2.7.1.69) are located in different regions of the chromosome and support different putative functions for the isoforms in relation to the effect of pressure. A similar observation has been made with isoforms of glyceraldehyde-3-phosphate dehydrogenase (EC:1.2.1.12) where one isoform (PBPRA2208) with 339 aa is up-regulated at 28 MPa and another isoform (PBPRA2602) with 330 aa is up-regulated at 0.1 MPa.

Under high pressure, the enzyme alcohol dehydrogenase (PBPRA2519), which converts acetaldehyde into alcohol, was found to be up-regulated. This suggests that the biochemical pathway responsible for the conversion of pyruvate into 2-Hydroxy-ethyl-ThPP is being activated. Interestingly, this observation implies that *P. profundum* may assume a fermentative metabolic phenotype under high pressure. How this shift in metabolism allows for cell survival under high pressures should be further investigated.

**Figure 4:**
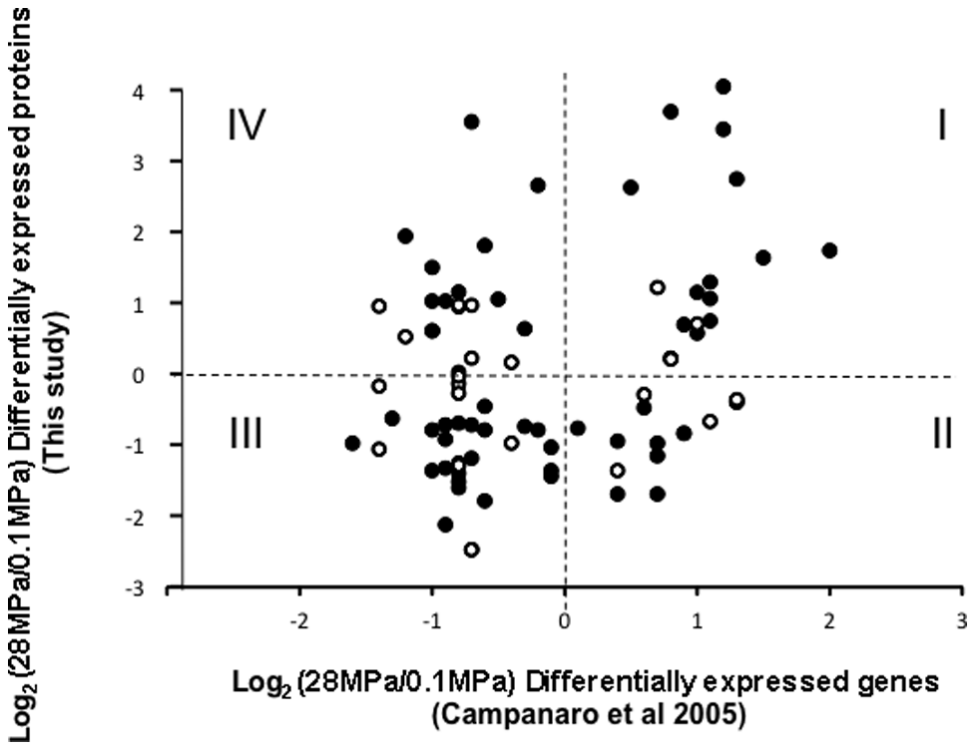
Correlation between the intensity ratio 28 MPa/0.1 MPa in log2 measured in this study at the protein level (y axis) and differentially expressed ORF 28 MPa/0.1 MPa in log2 presented in the study of Campanaro et al (2005) (ref. 4). The open circles are associated to proteins identified in this study with a p-value >0.05 and the filled circles are associated to proteins identified in this study having a p-value <0.05. We have only genes/proteins identified in both studies being significantly differentially expressed. We have divided the graphe into 4 quadrants (I,II,III,IV) where I and III are correlated while II and IV are anticorrelated.

Two comparative transcriptomic studies have been performed on *Photobacterium profundum* under different pressure regimes [4], [6]. In Figure 4, we compared the output from our current proteomic data with the published transcriptomic data. The overlap between the studies is only 82 proteins since the method of protein quantitation employed by each study is quite different. Empty circles represent the proteins identified in this study but with a low confidence quantitation level (p-value associated to the quantitation above 0.05). The filled circles are associated to those proteins, which were identified in this study and quantified with a p-value<0.05. In both cases, a trend between the proteomic and the transcriptomic study is observed (quadrant I and III contain 48 proteins). A possible mechanism for explaining anticorrelation between transcriptome (high ratio) and proteome (low ratio) is the presence of anti-sense RNA which could inhibit translation [48].

The presented dataset is too small to highlight meaningful trends in terms of protein function as highlighted by Hack [10]. That explains the observation of stress proteins having reciprocal trends in expression levels of mRNA (transcriptomic studies) versus protein (proteomic studies). The overall observations made in this study are consistent with the observations reported by other studies of *P. profundum* [10]–[12].

## Conclusions

We have analysed the proteome of *Photobacterium profundum* under different pressure regimes using a label-free quantitative proteomic analysis. An important fraction of this proteome is under tight regulation, with relatively highly abundant proteins being up- or down-regulated in function of the pressure. The data acquired in this study suggests that drastically altered modes of protein function exist under the different pressure regimes. As mentioned in other studies [4], [6], the difference in marine environments is not only characterized by a fundamental physical differences (i.e., pressure, light and temperature) which can play an important role in protein assembly and transport, but they represent completely unique ecological niches. By using the same growth medium in both pressure conditions, we highlighted that nutrient intake by *P. profundum* is potentially modulated by pressure.

Several of the differentially expressed proteins have been previously identified as playing important roles in cellular adaptation to altered atmospheric pressure. However, some of the differentially expressed proteins either have not previously been identified in high-pressure adaptation mechanisms or were not regulated as expected.

The increase in the number of new organisms being sequenced provides the opportunity for new proteomics studies to be generated. To our knowledge, we are reporting one of the first proteomic studies on *P. profundum*, a key model organism for understanding pressure adaptation and may have a valuable role in industrial and biotechnology applications.

## Supporting Information

Supplementary information S1 **Table of the complete list of proteins identified in the present study.**
(XLS)

Supplementary information S2 **Selected spectra of the proteins identified with only 1 unique peptide. Table and MS/MS spectra.**
(PPTX)

Supplementary information S3 **Kegg pathway enrichment using KOBAS for the proteins differentially expressed in** ***P. profundum*** **.**
(XLS)

Supplementary information S4 **Proteins significantly differentially expressed identified in this study, which are involved in the glycolysis/gluconeogenesis pathway according to Kegg.**
(PPT)
