# Supplementary material for: Photobacterium profundum under Pressure: A MS-Based Label-Free Quantitative Proteomics Study
Source: PLoS One. 2013 May 31;8(5):e60897. doi: 10.1371/journal.pone.0060897 (PMC3669370; doi:10.1371/journal.pone.0060897)
Supplement: Supplementary information S2 — Selected spectra of the proteins identified with only 1 unique peptide. Table and MS/MS spectra. (PPTX) [file pone.0060897.s002.pptx]

## Slide 1
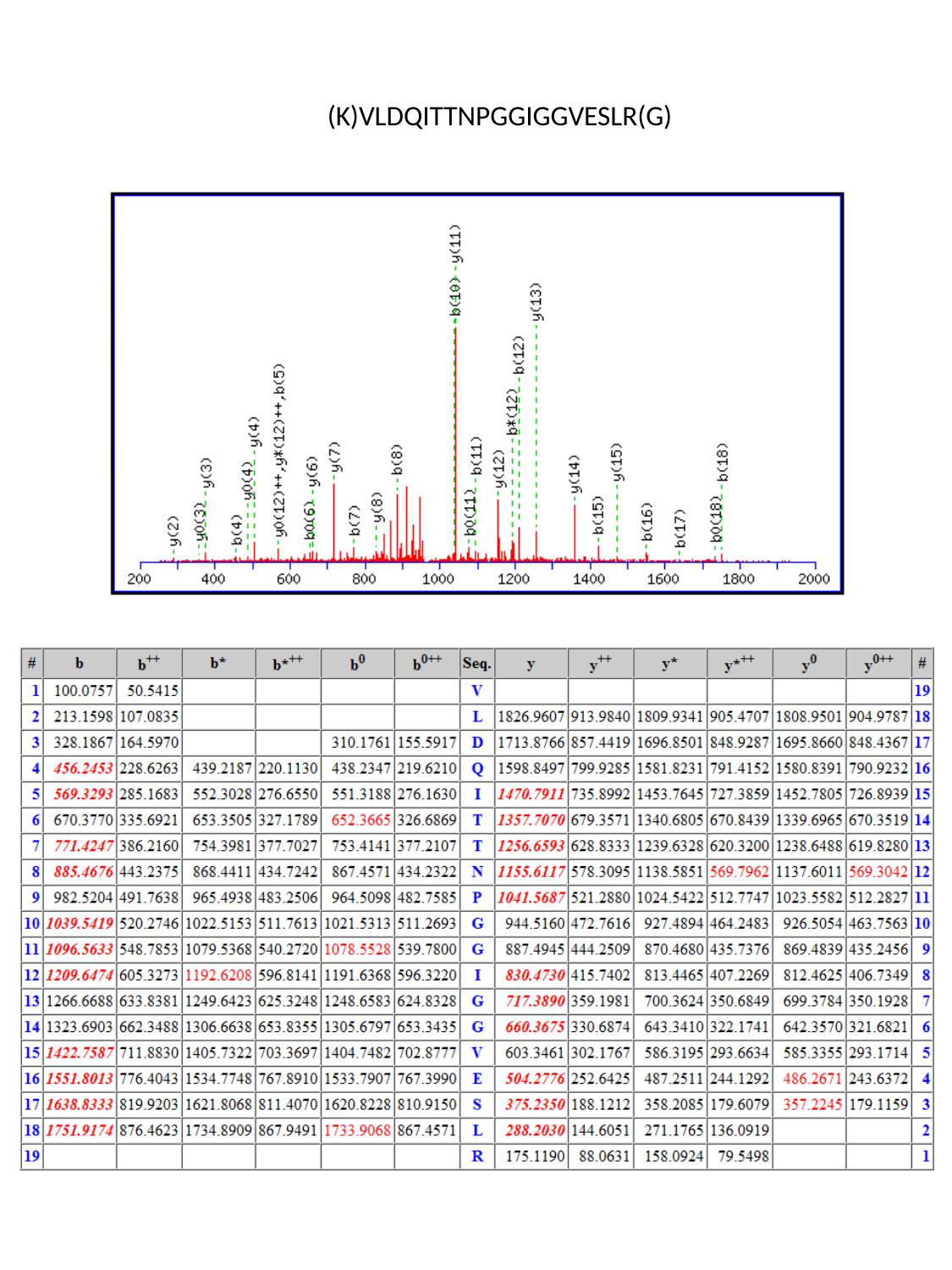

| (K)VLDQITTNPGGIGGVESLR(G) |
| --- |

## Slide 2
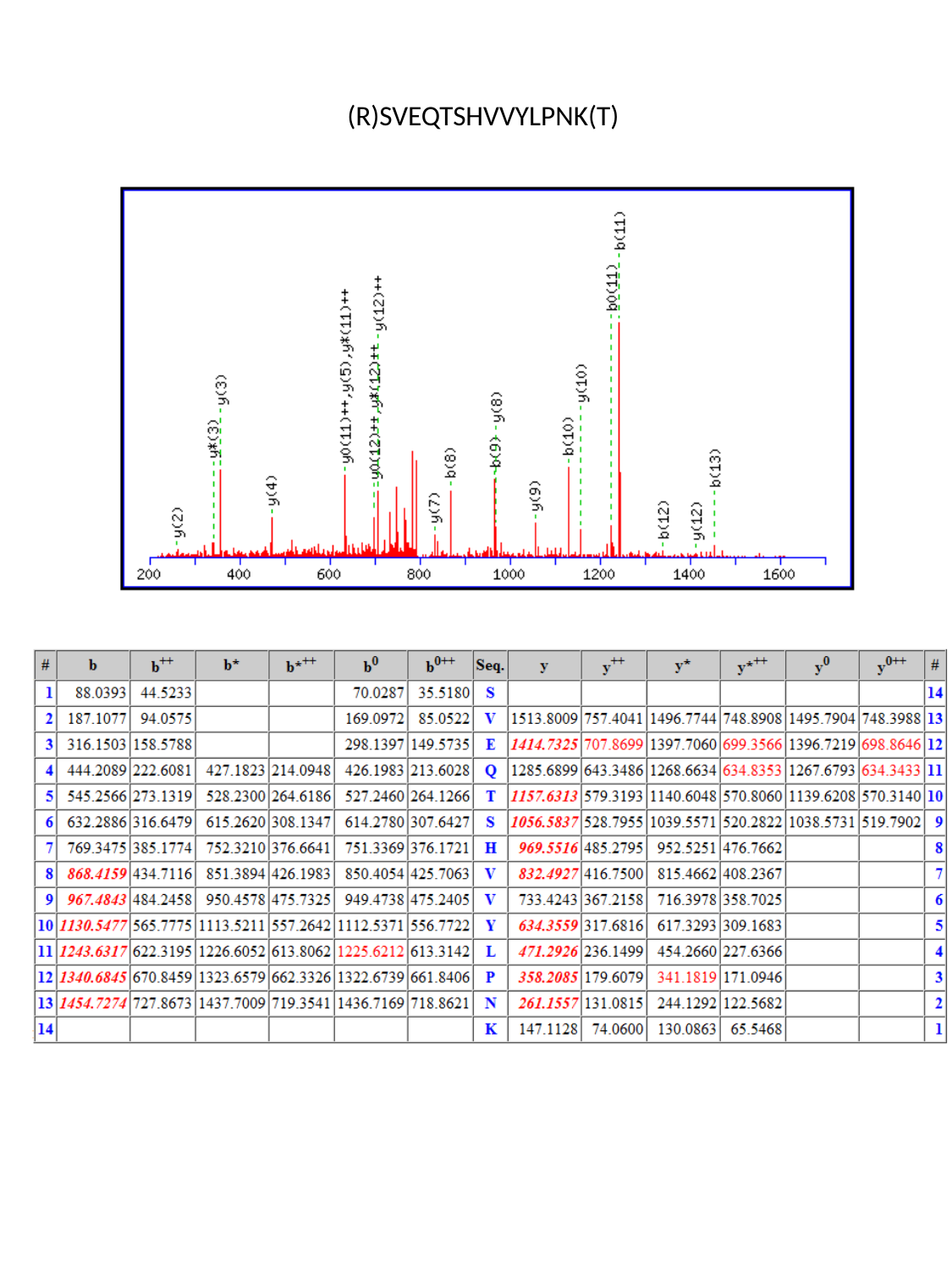

| (R)SVEQTSHVVYLPNK(T) |
| --- |

## Slide 3
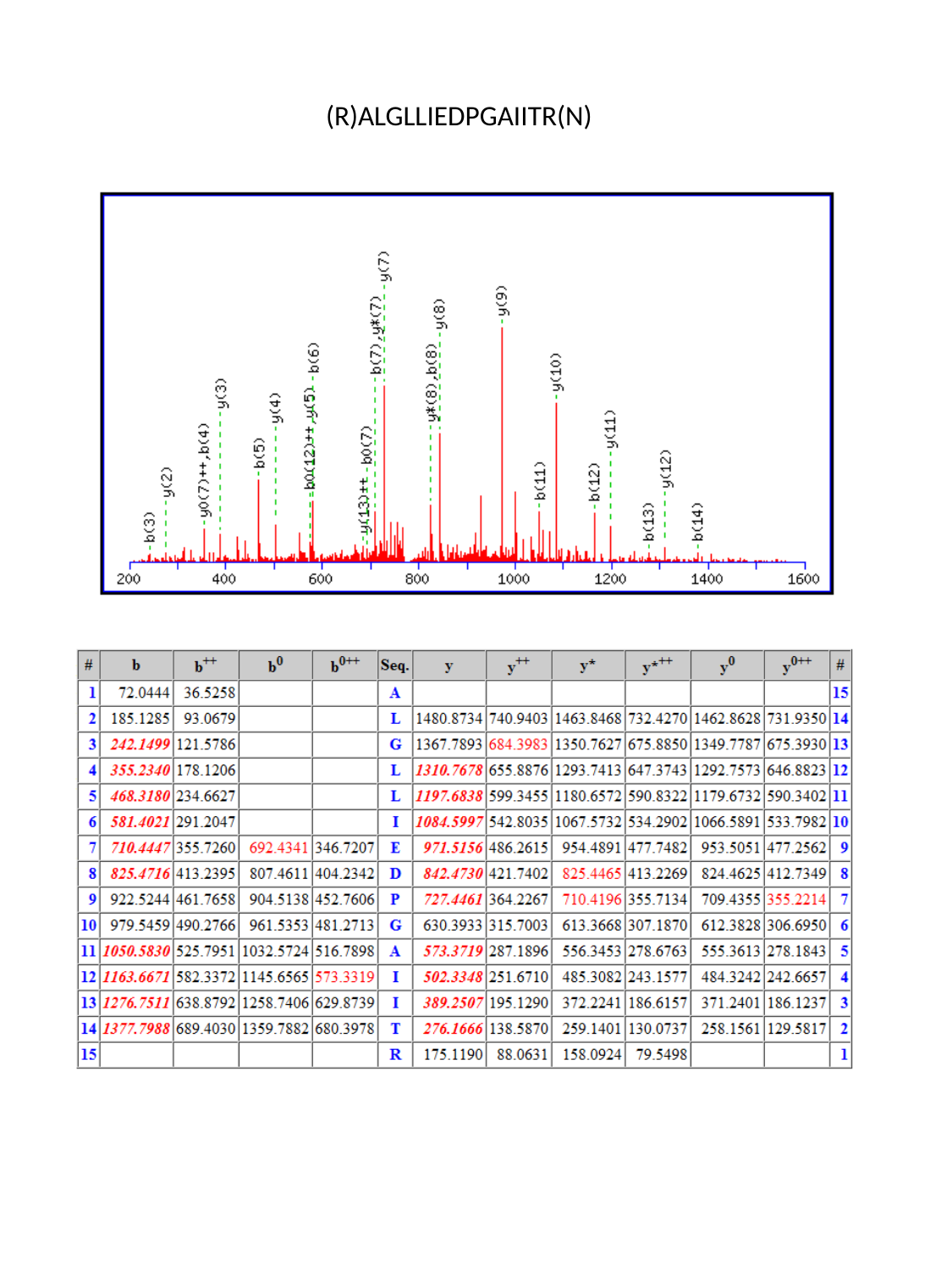

| (R)ALGLLIEDPGAIITR(N) |
| --- |

## Slide 4
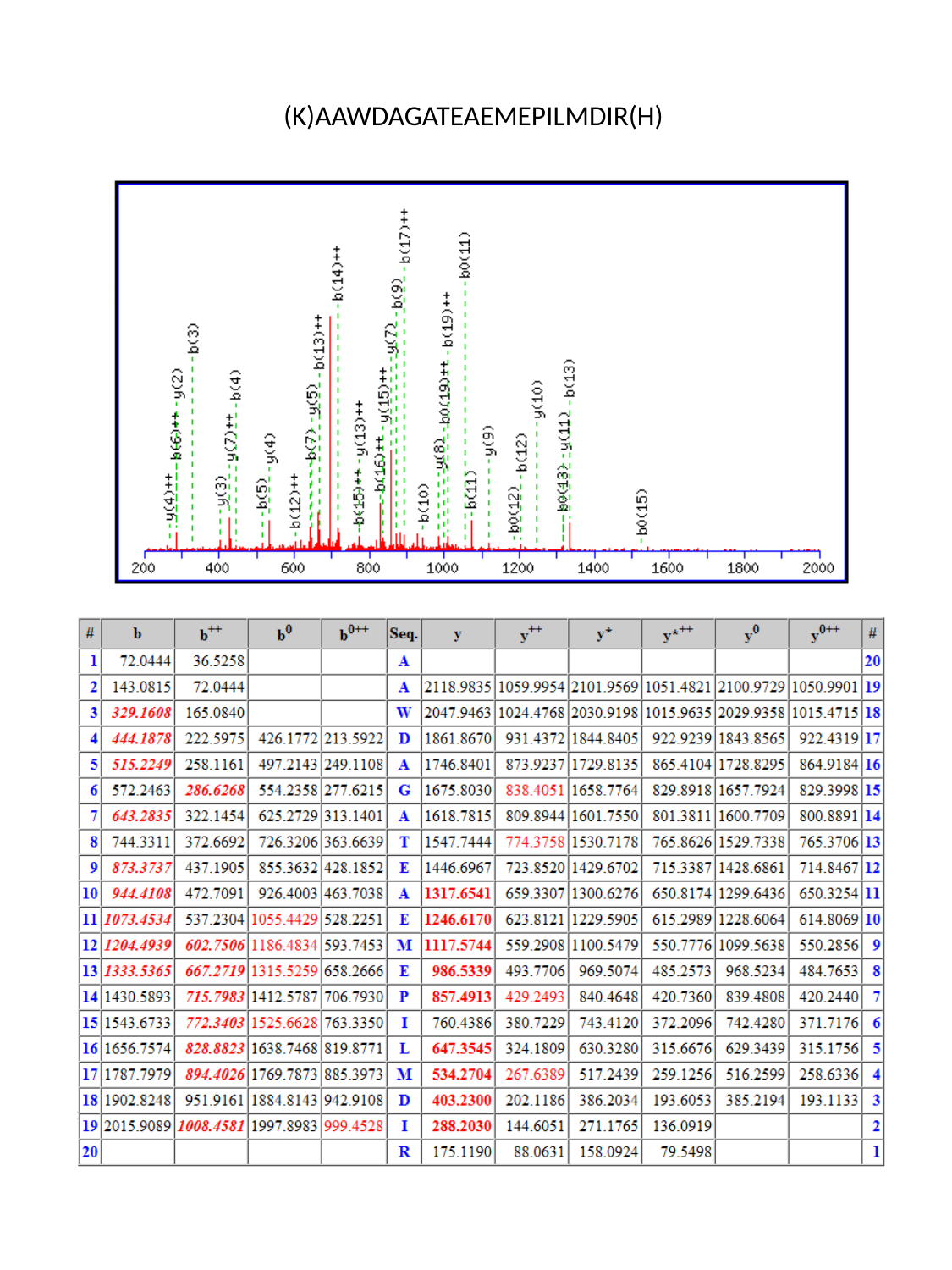

| (K)AAWDAGATEAEMEPILMDIR(H) |
| --- |

## Slide 5
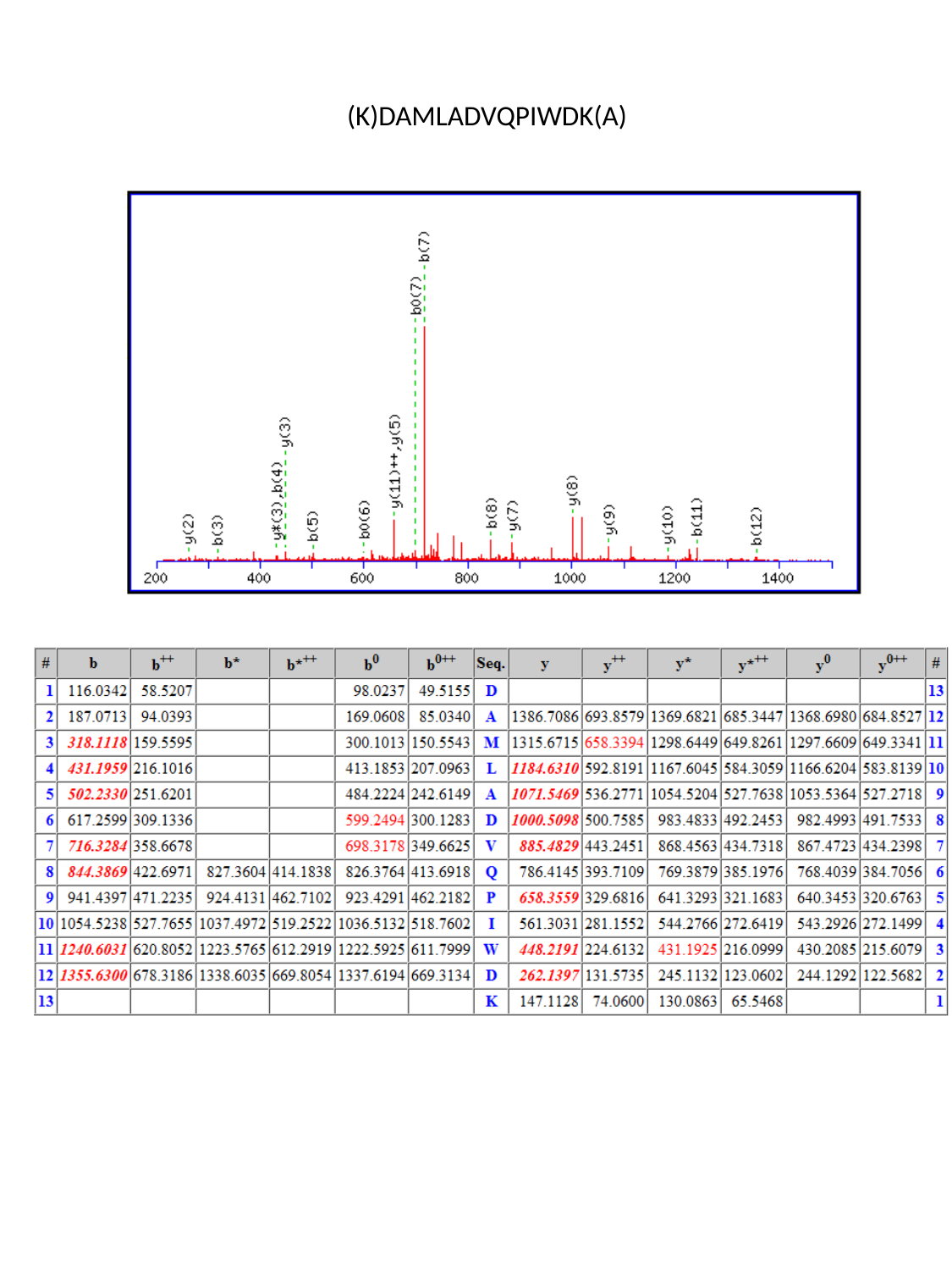

| (K)DAMLADVQPIWDK(A) |
| --- |

## Slide 6
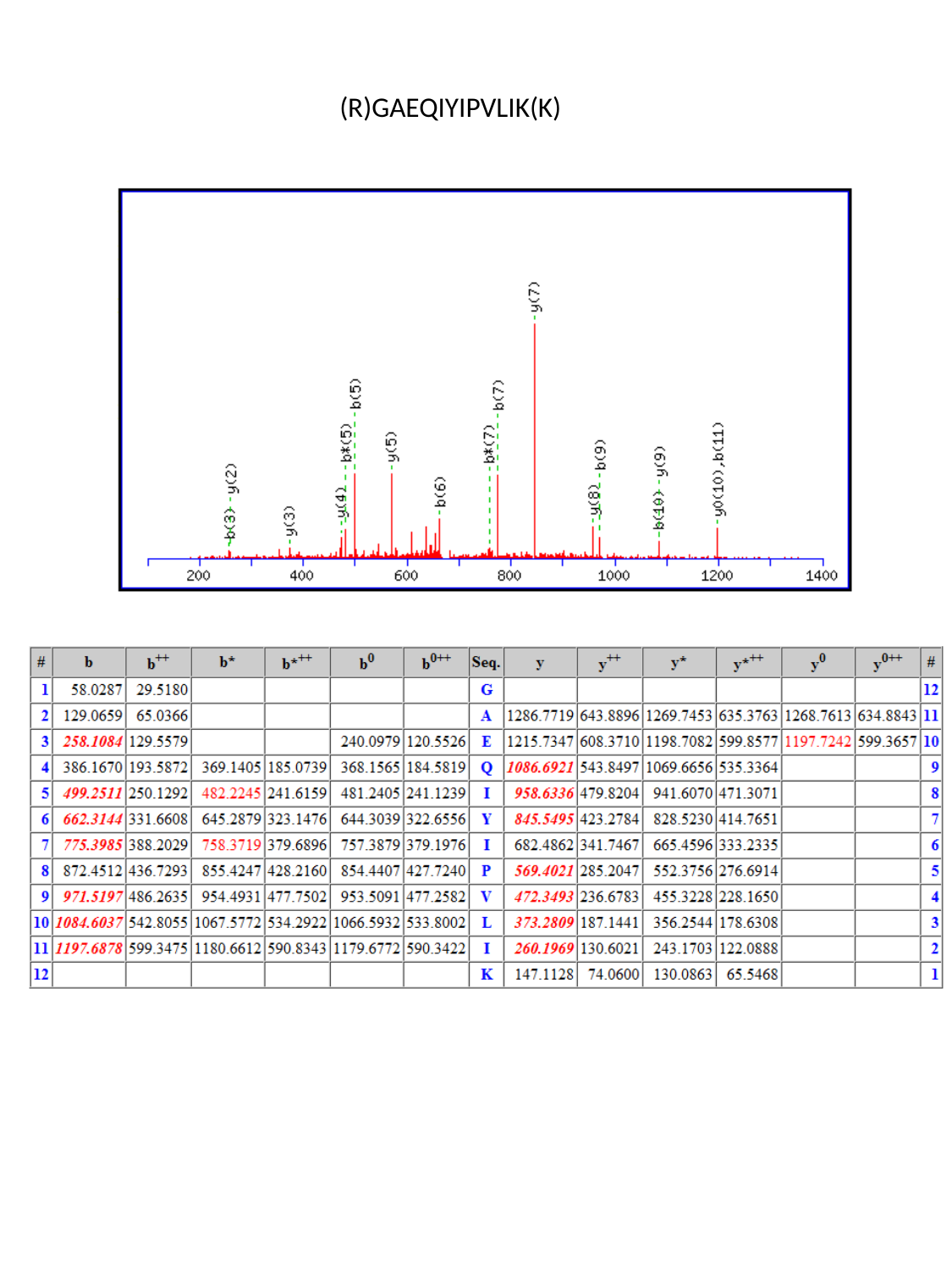

| (R)GAEQIYIPVLIK(K) |
| --- |

## Slide 7
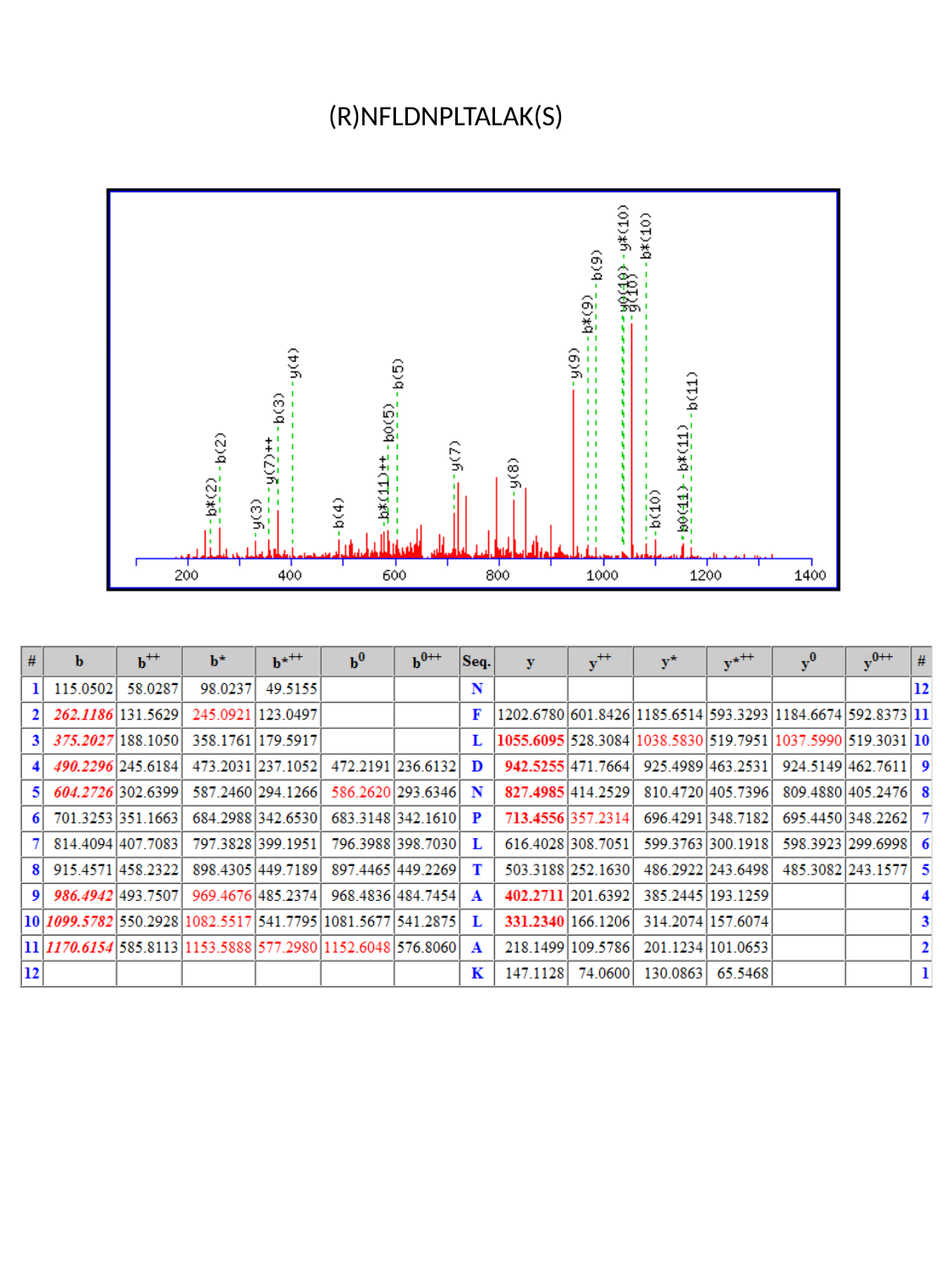

| (R)NFLDNPLTALAK(S) |
| --- |
