# Supplementary figures and images for: Photobacterium profundum under Pressure: A MS-Based Label-Free Quantitative Proteomics Study
Source: PLoS One. 2013 May 31;8(5):e60897. doi: 10.1371/journal.pone.0060897 (PMC3669370; doi:10.1371/journal.pone.0060897)

## Slide 1
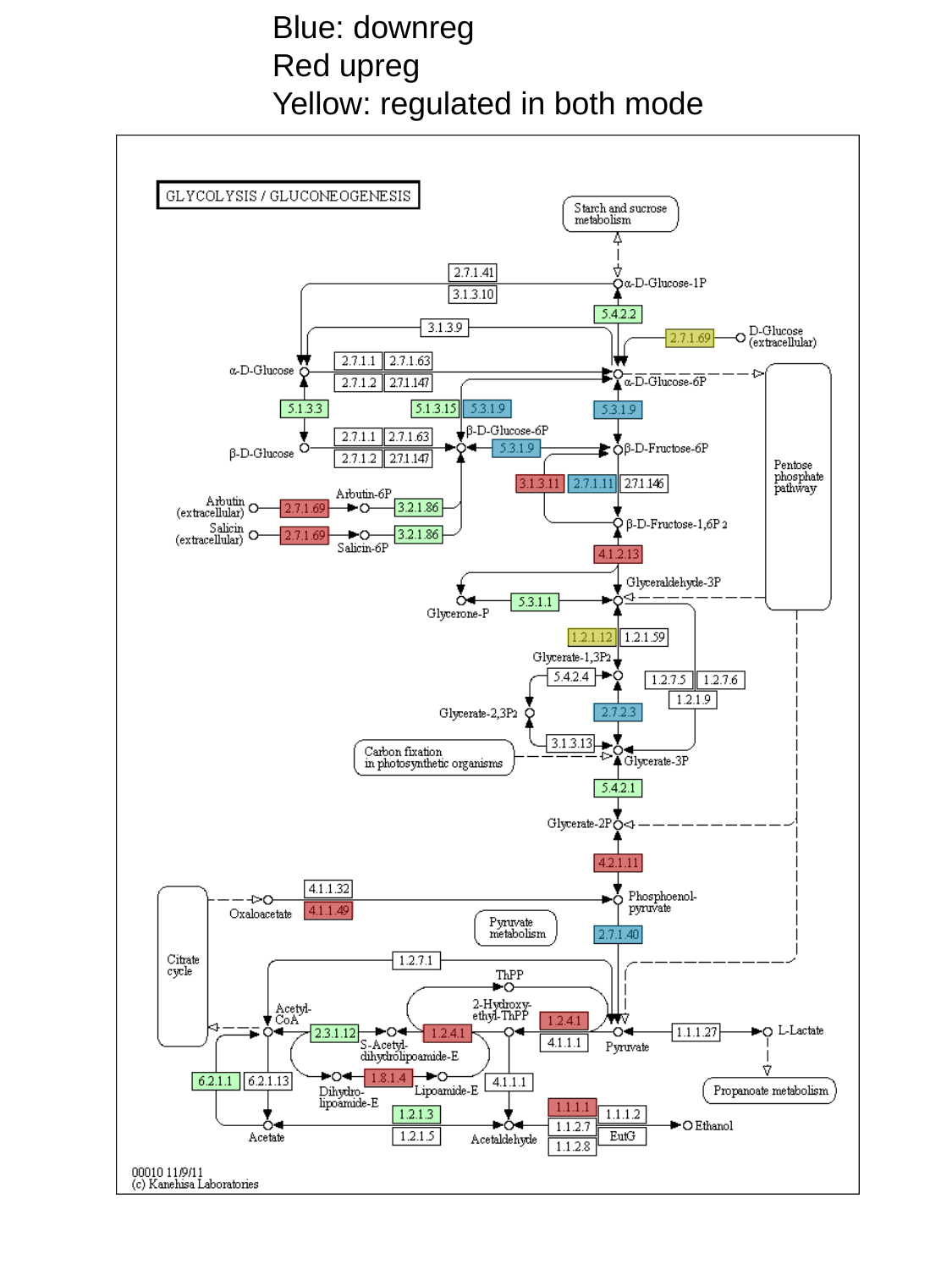

Blue: downreg
Red upreg
Yellow: regulated in both mode

Supplement: Supplementary information S4 — Proteins significantly differentially expressed identified in this study, which are involved in the glycolysis/gluconeogenesis pathway according to Kegg. (PPT) [file pone.0060897.s004.ppt]
